# What Factors Help Young Children Develop Positive Perceptions of Their Motor Skills?

**DOI:** 10.3390/ijerph18020759

**Published:** 2021-01-18

**Authors:** Lisa M. Barnett, Jill A. Hnatiuk, Ninoshka D’Souza, Jo Salmon, Kylie D. Hesketh

**Affiliations:** 1 Institute of Physical Activity and Nutrition, School of Health and Social Development, Deakin University, Geelong, VIC 3220, Australia; 2 Institute of Physical Activity and Nutrition, School of Exercise and Nutrition, Deakin University, Geelong, VIC 3220, Australia; jill.hnatiuk@deakin.edu.au (J.A.H.); n.dsouza@deakin.edu.au (N.D.); jo.salmon@deakin.edu.au (J.S.); kylie.hesketh@deakin.edu.au (K.D.H.)

**Keywords:** motor competence, physical self-perception, physical activity

## Abstract

A positive perception of motor skills is important for physical activity participation. The aim was to investigate which modifiable factors predict children’s perceived motor skills. Mothers completed questionnaires when their child was 3.5 and 5 years old. At 5 years old, the children’s perceived motor competence (PMC) was assessed. Separate linear regression models (up to 300 children) examined which factors at each time point predicted children’s PMC, adjusted for relevant confounders. Multivariate models were then run with factors associated (*p* < 0.10) with perception. At 3.5 years, the time spent with same age and older children (both higher tertiles) and parental physical activity facilitation (sum of facilitation in last month, e.g., taking child to park) were initially associated with higher perception. Dance/gymnastics participation were associated with lower perceptions. Other child behaviours, maternal beliefs, play equipment, and swimming lessons were non-significant. In the final prospective model (*n* = 226), parental physical activity facilitation when child was 3.5 years old was the only factor to predict PMC. No factors were significant for the cross-sectional analyses at 5 years. Perceptions are formed based on past experiences which may explain why factors at 3.5 years rather than current experiences (when children were 5 years old) were associated with childhood perceptions.

## 1. Introduction

The most recent World Health Organisation guidelines (released in 2019) recommend children aged 3 to 4 years old should spend at least 180 min (three hours) per day in a variety of physical activities, with at least one hour of this time in moderate- to vigorous-intensity physical activity (MVPA). These recommendations have arisen because of the considerable benefits physical activity can provide. For example, a systematic review conducted for the 2018 Physical Activity Guidelines Advisory Committee Scientific Report highlighted physical activity was associated with better bone health and better weight status in children aged 3 to 6 years old [1]. Yet, only 6 in 10 (61%) Australian children aged 2–5 years met the Australian version of this recommendation (physically active each day for at least three hours, with at least 60 min ‘energetic play’) [2]. Therefore, understanding the correlates of physical activity in young children is important for informing the development of future strategies to increase participation.

Positive physical self-perception is a broad umbrella concept that includes the perception of factors such as strength, fitness, balance, flexibility, body attractiveness, and sport competence [3,4]. Physical self-perception has been reported as a key correlate of physical activity participation in older children and youth [5]. Based on the competence motivation theory of Harter [6], children who are provided with the opportunity to practice their skills and achieve mastery will develop positive feelings about their physical competence.

Self-perception in motor skill competence (i.e., how well a child thinks they can run, jump or throw) and active play skills (e.g., cycling) are specific components of physical self-perception relevant to children and situated within the broader factor of perception of sport competence [7]. Perceived motor competence (PMC) is highlighted as a mediator between actual motor competence and physical activity in the conceptual model by Stodden et al. [8]. This model is developmental, in that the directionality within the constructs in the model change as children age, i.e., younger children engage in physical activity which in turn develops their motor competence, and as they age, this increased motor competence translates back to higher physical activity levels. Recently, the relationship between actual and PMC in children was examined via a meta-analysis in 54 studies, with a small significant positive effect reported for overall motor competence (*r*  =  0.25) [9]. Thus, whilst the relationship between these variables is confirmed, it is not strong, suggesting that different factors drive children’s PMC, rather than their actual competence. As such, it is important to understand drivers of perception as separate to actual motor competence.

Two longitudinal studies support the proposed pathway directions in the Stodden et al.’s model in relation to PMC. In the first, with data from the same study as the current paper, the MVPA of children aged 3.5 years was positively associated with PMC at 5 years of age [10] and in the second study, 8 year old children with higher PMC, had higher MVPA one year later [11]. One study investigating active play skill perception (e.g., cycling), reported perception was positively associated with physical activity in school age, but not preschool age children [12], also suggesting these relationships may strengthen across development. However, physical activity opportunities may not always enhance perception. For instance, learning to swim can take some time and a child who attends lessons may experience reduced perceptions of competence due to a rising awareness of how much further there is to go before they become a competent swimmer. Thus, self-perception is affected by development. When a child reaches middle childhood, or around eight years old, they tend to become more cognitively astute and realistic about their abilities and how they compare with peers [13]. Theoretically, this can mean their perception decreases [13]; although this is not always supported in the literature [14]. Nevertheless, in young children (<8 years old), positive self-perception of motor skill competence is important to develop as this will contribute to further engagement in physical activity [8].

Horn [15] recommends focusing research attention on young children because this is when perceptions of competence are developing and when significant adults have a large impact on the physical activity of children. Thus, it is important to understand more about what factors help young children to develop positive motor skill perceptions. Positive feedback from significant others, such as parents and teachers, is an important part of the development of physical self-perception, and helps children experience pleasure in developing competence [15]. Thus, mothers who believe they can encourage their children to be active, and are confident they can provide physical activity opportunities, might have children who develop more positive motor skill perceptions.

The physical activity opportunities parents provide their children might also influence their children’s motor skill perceptions. This might involve facilitating children’s physical activity, providing a home environment with adequate play equipment for physical activity and/or enrolling their child in organised activities. A large recent Finnish cross-sectional study (*n* = 472) of six-year-old children reported organised sport participation was associated with higher object control skill perceptions and less time in sedentary behaviour was associated with higher locomotor skill perceptions [16]. In organised physical activities, children will often participate with peers of a similar age. This interaction with peers is another way a child develops an understanding of their own motor skill competence. Playing with other children, particularly older children, was reported in a cross sectional study to encourage role modelling of skill performance [17]. Seeing how another child perform skills also assists a child to understand where they are in their own development.

Thus, whilst it is clear that interaction with peers and feedback from significant adults can assist children to develop perceptions of their own capabilities, there is limited longitudinal evidence regarding what modifiable factors can assist children to develop positive motor skill perceptions. The aim of this study was to investigate which modifiable family and home environment factors (child behaviours, maternal beliefs, parental behaviours, home environment, organised activity participation) were associated with children’s PMC. We hypothesised that physical activity opportunities in the home and community, and maternal beliefs and parental support for physical activity at 3.5 years and 5 years would be positively related to children’s PMSC at 5 years.

## 2. Materials and Methods

Data were drawn from the Melbourne Infant Feeding, Activity and Nutrition Trial (InFANT) Program Follow-Up. The original Melbourne InFANT Program was a 15-month obesity prevention intervention (2008 to 2010) targeted to first-time parents and their babies aged 4 and 19 months of age [18]. Post-intervention follow-up occurred when the child was aged 3.5 years (2011–2012) and 5 years (2013) [19]. The original intervention contained 542 parent–child pairs (86% response rate) from 62 different child-health centre parent groups. Four-hundred eighty (89% retention) families were still enrolled by the end of the intervention. The analyses in this paper do not report on intervention outcomes, but intervention status is controlled for, given the original study design, despite no differences in PMC observed between groups (data not shown).

Data for the current study were drawn from 305 children. Parents completed pen and paper questionnaires at both time points, and children completed PMC assessment at 5 years only. Ethical approval to conduct the study has been granted by the University Ethics Committee (EC 175-2007) and by the Victorian Office for Children. Parents gave written informed consent for themselves and their children.

Demographic information was collected at the baseline point of the intervention (child aged 4 months old) via the written questionnaire from the responding parent (all mothers), including maternal country of birth (Australia/Other), highest level of maternal education (Low = secondary school or less; Medium = trade certificate/diploma; High = University qualification), and maternal and child date of birth (to calculate age) and child sex.

The questionnaire also asked parents about child and family factors hypothesised to be correlates of PMC. These included child behaviours (e.g., time spent: being physically active with mum, free to move around (i.e., not restrained), with other children of a similar age, with older children, and outside), maternal beliefs (e.g., maternal physical activity knowledge, views, optimism and self-efficacy), parental behaviours (e.g., facilitation of physical activity), and the home environment (e.g., ball skill equipment and play equipment available in home) and community environment (e.g., participation in organised sport). Processing of the child behaviours, maternal beliefs, parental facilitation, and home environment variables at 3.5 years and 5 years was consistent with previous research with this sample at 3.5 years of age [20]. In brief, child behaviours, reported in average minutes per week, were divided into tertiles to account for skewed data; capping of time spent ‘free to move about’ and ‘with other/older children’ occurred at 14 h/day and time ‘outdoors’ and ‘being physically active with mum’ at 7 h/day [20]. Maternal beliefs were assessed on a 4-point Likert scale (0 = strongly disagree/not at all confident to 3 = strongly agree/extremely confident). Average scores for each belief (knowledge = 8 items at 3.5 years old (α = 0.75) and 7 items at 5 years old (α = 0.71); views = 4 items (child aged 3.5 years α = 0.70, child aged 5 years α = 0.68); optimism = 3 items (child aged 3.5 years α = 0.82, child aged 5 years α = 0.86); self-efficacy = 3 items (child aged 3.5 years α = 0.79, child aged 5 years α = 0.82) were calculated. Parent facilitation comprised 7 items rated on a 6-point Likert scale assessing frequency of parents facilitating physical activity for their child (e.g., taking them to the park, for a bike ride, etc.), converted into weekly equivalent scores (possible score range 0–98) (child aged 3.5 years α = 0.80, child aged 5 years α = 0.81). Home equipment was assessed on a 7-point Likert scale assessing how often the child uses the equipment item at home. Ball skills equipment (*n* = 2 items; possible score range 0–14) and play equipment (*n* = 7 items; possible score range 0–49) were separated as differences have been found according to skill type opportunities (e.g., object control) when investigating actual motor competence [21]. Type of organised activity participation (up to five activities) was freely reported by mothers in the questionnaires at 3.5 and 5 years. Data were subsequently classified as participation (Y/N) in ‘sport’ (comprising ball skill related sport [e.g., soccer, tennis, variety of mini sport sessions), dance/gymnastics, or swimming. Organised activities reported by parents that could not clearly be identified as relevant to movement skill development (e.g., music, language classes) or had too few children (<5%) participating in them (e.g., martial arts) were omitted from analyses. All constructs have shown adequate test-retest reliability in preschool children (29).

The children’s PMC was assessed using the pictorial scale of Perceived Movement Skill Competence [22,23]. The children were shown 12 skill items, six locomotor (run, gallop, hop, leap, horizontal jump, and slide) and six object control skills (striking a stationary ball, stationary dribble, kick, catch, overhand throw, and underhand roll), that were matched to the Test of Gross Motor Development- 2nd Edition (TGMD-2) [24]. A further six items assessed play skill competence (bike riding, scootering, paddling on a board, climbing a rope, skating/blading, swimming) [23]. Each item had two cartoon images, one of the child performing the skill well and one of the child not performing the skill. Boys were shown images of boys and girls were shown images of girls. The children were told—‘This boy is very good at running, this boy isn’t very good at running, which boy are you like when you run?’ Then, the children were asked, (if they chose the better performance), ‘Are you really good at running or pretty good?’, or if they chose the poorer performance, ‘Are you not too good at running or sort of good?’. The result was a four-point scale for each item. The PMSC has good reliability [22] and construct validity [23] in Australian young children. In this sample, the internal consistency for the 18 PMC items was excellent (α = 0.85).

Data were analysed using Stata v.15.0. Proportions and means were derived as descriptive statistics. Tests of differences (i.e., t tests or difference in proportions) between predictor variables (child behaviours, maternal beliefs, parental behaviours, home environment, community environment,) assessed at 3.5 y and 5 y were conducted. Main data analyses followed a two-stage process. First, univariate associations between each predictor variable at each time point and the outcome variable (PMC) was examined using linear regression models. Any predictor variables that showed an association of *p* < 0.10 were then entered into a separate multivariate model for the relevant time point. Effect size was estimated using Cohen’s f^2^ (f = 0.10; small effect, f = 0.25; medium effect, f = 0.40; large effect) for individual predictors included in the multivariate model. All models controlled for the child age and sex, the original intervention group, and the cluster-based recruitment method.

## 3. Results

The sample characteristics are presented in Table 1. Around half the mothers were university educated and most were born in Australia.

The descriptive data for each of the predictors at 3.5 y and 5 y are presented in Table 2 as means (standard deviations), proportions (% yes/no), or the tertile cut points. When comparing predictors at 3.5 and 5 y, the following were different at time 2: time spent being physically active with mum (lower), time spent with children of a similar age (higher), maternal physical activity knowledge (lower), maternal physical activity views (higher), maternal physical activity optimism (lower), parental facilitation of physical activity (lower), ball skills equipment in the home (lower), sport participation dance/gymnastics and swimming (all higher).

Table 3 reports associations between the predictors at 3.5 years and children’s PMSC at 5 years. For the univariate predictor models, children who spent the most time (highest tertile) with other children of a similar age and with older children had higher PMSC than children in the lowest tertile for both predictors. Further, parental facilitation of physical activity was positively associated with PMSC. Children who participated in dance/gymnastics displayed lower PMSC than those who did not. For the multivariate prospective models, only parental facilitation of physical activity at 3.5 years was positively associated with PMSC. All other child behaviours, maternal physical activity beliefs (knowledge, views, optimism and self-efficacy), home and community environment predictors were non-significant.

Table 4 reports cross-sectional associations between the predictors and PMSC at 5 y. None of the predictors were cross-sectionally associated with PMSC for the univariate models (thus multivariate models were not tested for).

## 4. Discussion

The aim of this study was to understand which modifiable family and home environment factors were associated with children’s PMC, both longitudinally (from 3.5 to 5 years old) and cross-sectionally (at age 5 years). In the final prospective model, parental physical activity facilitation (sum of facilitation in last month, e.g., taking child to park, for a walk, for a bike/scooter ride) was the only factor indicated to be a significant positive predictor of children’s movement skill perception at 5 years old, after accounting for other predictors. In the cross-sectional analyses at 5 years old, no factors were associated with children’s movement skill perceptions. One other study noted that self-perceptions in 5 year old children are formed based on experiences up to that age [25], which may explain why current experiences were not associated with childhood perceptions in this study. Thinking more broadly than physical activity perception, the parental role and relationship with their child in the early years is important for establishment of child self-esteem [26], and these experiences prior to age 5 years are likely to prospectively influence motor skill perceptions.

The findings support that time spent with same age and older children and parental facilitation are key predictors of child movement perceptions—when the child was 3.5 y. Time spent with other children of a similar age was significantly higher at 5 y compared to 3.5 y and parental facilitation of physical activity was lower, which might help to explain why these variables were not predictors at 5 y. A separate analysis of these data, which examined predictors of actual motor competence, reported more home physical activity equipment when child was aged 3.5 years was predictive of better object control skill at age 5 years [20]. Therefore, to develop actual movement skill competence, environmental opportunity is important, whereas developing a positive perception of motor competence may relate more to the quality of interactions with significant others in movement contexts.

In the prospective models, factors related to interaction with others (such as time with same age and older children), were predictive of higher perceptions. This could be because children who play more with others have more opportunity to benchmark their skill level with other children; although speculatively it might be expected that this would result in a negative self-assessment (rather than positive) in reference to older children’s greater capabilities. Alternatively, children who spend more time playing with older children may develop better motor skills, which over time results in higher self-perceptions. If children have supportive siblings who encourage their performance, however limited, this might also contribute to higher perceptions. The nature of the feedback and interaction around movement experiences can create different mindsets in children. In short, a child with a growth mindset believes that they can do better with further effort, whereas a child with a fixed mind set thinks they cannot develop further. Haimovitz and Dweck [27] suggest that process-oriented, (e.g., “you worked out a great way to leap further”), rather than person-focused praise, (e.g., “you are a great leaper!”), is integral to a child developing a growth or learning mindset.

The notion that more time spent playing with older children may develop better motor skills, was supported in a separate analysis of these data, as time with older children (when children were aged 3.5 years) was predictive of actual locomotor skills [20]. In this study we did not separate perception into skill domains (e.g., object control and locomotor) as it was not possible to clearly distinguish the predictors in terms of how they might feasibly relate to either locomotor or object control perception (e.g., time outside would be hypothesised to relate to both domains of perception). Other literature in this area has not investigated interaction with peers as a potential correlate of children’s movement skill perception [16].

One study in Belgian preschool children, which examined parental interaction as a potential correlate of actual movement skills, found a trend for significance in girls, where greater father–child interaction in creative and dance activities was associated with *lower* movement skills [28]. In the current study, children’s participation in organised dance/gymnastics at age 3.5 years was initially associated with lower movement perceptions at age 5 years. The time involved in dance or gymnastics may take away from time in other movement pursuits that would contribute to developing a positive overall perception of movement competence. This notion is supported by an Australian cross-sectional study investigating correlates of actual motor skill in pre-schoolers that found dance was associated with lower object control (ball related) skills, with this finding driven by the girls in the sample [29]. Australian girls more often participate in activities such as dance and gymnastics, compared to boys [30], and girls are also reported to have lower actual object control skills [21] and lower object control skill perceptions [23]. Although, in the cross-sectional analyses, more children participated in dance/gymnastics but there was no relationship with movement perceptions.

The main strength of this study is the novelty of the investigation, as few studies have investigated socioecological predictors of children’s movement skill perceptions, particularly longitudinally. Another strength is the broad range of potential predictors and the robust measure of PMC. It is important to note that whilst a number of predictors when the child was 3.5 years were significant, that effect sizes were small. Proxy report by parents was limited, in that the time spent in certain behaviours is only indicative. Another limitation was that this cohort was originally powered for an intervention and as such, the sample used in this paper may not have been powered for the current analyses. Finally, the sample may not be generalisable, as around half the mothers were University educated and most were born in Australia. Future researchers may wish to investigate other potential predictors of motor skill such as nutritional status and socio-economic status.

## 5. Conclusions

Our results suggest the role of parents in facilitating children’s activity helps children understand their own movement performance. Our lack of significant findings in the final models also show that other unmeasured factors are contributing to children’s perceptions, or simply that past experiences, rather than present experiences are most important to children’s current perceptions. Future research could continue to explore which factors help to develop children’s movement perceptions, so that we can ensure young children form perceptions that will enable them to engage in a lifetime of physical activity. We know that physical self-perceptions are an important correlate of physical activity in their own right, and that whilst PMC is associated with actual motor competence it is not a strong association, confirming that both can contribute in different ways to physical activity behaviour.

## Figures and Tables

**Table 1:** Demographic characteristics of the sample (children with any predictors and perception data at 5 y).

	*n*	Mean (SD) or %	Range
Child characteristics			
Age at 3.5 y	289	3.6 (0.19)	3.2–4.3
Age at 5 y (5 missing with no age data)	300	5.0 (0.12)	4.8–5.7
Sex	305		
Boys	161	52.8%	-
Girls	144	47.2%	-
Maternal characteristics			
Maternal education	304		
Low (Secondary school or less)	55	18.0%	-
Medium (Trade certificate/diploma)	70	23.0%	-
High (University degree and above)	180	59.0%	-
Country of birth	304		
Australia	254	83.6%	-
Other	50	16.4%	-
Perceived motor skill total score at 5 y	305	61.5 (7.7)	31–72

**Table 2:** Description of predictors included at 3.5 y and 5 y.

Variable	Description and/or Coding	Child Aged 3.5 y Mean (SD) or Tertile Cut-Points	Child Aged 5 y Mean (SD) or Tertile Cut-Points
**Child behaviours (time in minutes)**			
Time spent being physically active with mum	Proxy-report of the time spent in the last week. ^1,^** Data divided into tertiles (low,medium,high), due to the distribution of the data.	Low = 0–420 Medium = 435–840 High = 860–2850	Low = 0–330 Medium = 340–660 High = 690–2580
Time spent ‘free to move around’	Proxy-report of the time spent in the last week Data divided into tertiles (low,medium,high), due to the distribution of the data	Low = 0–3360 Medium = 3480–5040 High = 5220–5880	Low = 0–3360 Medium = 3480–4620 High = 4680–5880
Time spent with other children of a similar age	Proxy-report of the time spent in the last week ^2,^** Data divided into tertiles (low,medium,high), due to the distribution of the data	Low = 0–660 Medium = 720–1740 High = 1800–5880	Low = 0–1410 Medium = 1440–2220 High = 2280–5880
Time spent with older children	Proxy-report of the time spent in the last week Data divided into tertiles (low,medium,high), due to the distribution of the data	Low = 0–0 Medium = 50–420 High = 465–5580	Low = 0–0 Medium = 30–270 High = 285–5760
Time spent outside	Proxy-report of the time spent in the last week Data divided into tertiles (low,medium,high), due to the distribution of the data	Low = 0–840 Medium = 870–1350 High = 1365–2910	Low = 0–840 Medium = 860–1260 High = 1290–2940
**Maternal beliefs**			
Maternal physical activity knowledge ^a^	Composite score (scale 0–3) of eight ^b^ questions examining the importance of physical activity for children’s health and development **	2.45 (0.32)	2.27 (0.34)
Maternal physical activity views ^a^	Composite score (scale 0–3) of four questions assessing mothers’ views of physically active children **	1.86 (0.46)	2.13 (0.46)
Maternal physical activity optimism ^a^	Composite score (scale 0–3) of three questions examining the anticipated ease of engaging children in physical activity *	2.30 (0.50)	2.19 (0.52)
Maternal physical activity self-efficacy ^a^	Composite score (scale 0–3) of three questions examining mothers’ confidence for promoting physical activity	2.35 (0.51)	2.30 (0.56)
**Parental behaviours**			
Parental facilitation of physical activity	Summed score of seven questions on parental facilitation of physical activity for children (1 Never or rarely, 2 Some days each week, 3 Most days each week, 4 Every day, 5 At least once a day, 6 Several times each day, converted into weekly equivalent scores) **	49.90 (10.13)	34.12 (13.92)
**Home environment**			
Ball skills equipment in home	Summed score of two seven-point questions (possible score 0–14) on how often the child uses balls skills equipment at home #	8.51 (2.64)	8.08 (2.48)
Play equipment in home	Summed score of seven x seven-point questions (possible score 0–49) on how often the child uses play equipment at home	22.84 (7.56)	22.72 (6.80)
**Community environment**			
Sport participation (Y/N)	Proxy-report of participation in various ball skill related sports per week? **	Yes = 9.2% No = 90.8%	Yes = 16.1% No = 83.9%
Dance/Gymnastics (Y/N)	Proxy-report of participation in dance/gymnastics per week? **	Yes = 15.7% No = 84.3%	Yes = 25.9% No = 74.1%
Swimming (Y/N)	Proxy-report of participation in swimming per week? **	Yes = 48.9% No = 51.2%	Yes = 65.9% No = 34.1%

Notes. # *p* < 0.10, * *p* < 0.05, ** *p* < 0.001 indicates significant differences between predictors at 3.5 y and 5 y. ^1^ Difference between time points conducted using mean values (minutes) at 3.5 y (706.39) versus at 5 y (571.14). ^2^ Difference between time points conducted using mean values (minutes) at 3.5 y (1443.16) versus at 5 y (2060.13), ^a^ Please refer to Hnatiuk et al., 2013 for further processing details; ^b^ Seven items at 5 y.

**Table 3:** Predictors of 3.5-year-old children’s perceived motor skill competence (PMSC) at 5 years old ^1^.

Variable	Univariate Models Sample Size (*n*)	Univariate Models β (95%CI)		Multivariate Model (*n* = 226) β (95%CI)	Effect Size f^2^
**Child behaviours ^2^**					
Time spent being physically active with mum	252	Medium High	0.98 (−1.24, 3.20) ^1^ −0.15 (−2.57, 2.23)	-	-
Time spent ‘free to move around’	219	Medium High	−0.52 (−3.06, 2.03) ^1^ 0.38 (−2.10, 2.87)	-	-
Time spent with other children of a similar age	242	Medium High	0.13 (−2.21, 2.48) ^1^ 2.65 (0.34, 4.97) *	−0.39 (−2.85, 2.07) ^1^ 2.00 (−0.57, 4.57)	0.0137
Time spent with older children	246	Medium High	1.62 (−0.66, 3.90) ^1^ **2.17 (−0.20, 4.54) #**^,1^	1.98 (−0.35, 4.31) ^1^ 1.84 (−0.80, 4.48)	0.0124
Time spent outside	260	Medium High	−1.35 (−3.61, 0.91) ^1^ 0.63 (−1.70, 2.97)	-	-
**Maternal beliefs**					
Maternal physical activity knowledge	260		−1.44 (−4.36, 1.48)	-	-
Maternal physical activity views	260		1.21 (−0.83, 3.26)	-	-
Maternal physical activity optimism	260		0.86 (−1.02, 2.75)	-	-
Maternal physical activity self-efficacy	260		−1.36 (−3.20, 0.47)	-	-
**Parental behaviours**					
Parental facilitation of physical activity	250		0.09 (−0.01, 0.19) *	0.11 (0.01, 0.21) *	0.0096
**Home environment**					
Ball skills equipment in home	255		−0.14 (−0.52, 0.23)	-	-
Play equipment in home	253		0.03 (−0.09, 0.16)	-	-
**Community environment**					
Sport participation (Y/N)	300		0.41 (−2.59, 3.40)	-	-
Dance/Gymnastics (Y/N)	300		Ref. (No) −2.45 (−4.84, −0.06) *	Ref. (No) −2.35 (−4.98, 0.28)	0.0136
Swimming (Y/N)	300		Ref. (No) 0.55 (−1.17, 2.28)	-	-

Notes. # *p* < 0.10, * *p* < 0.05, ** *p* < 0.001. All analyses controlled for child’s sex, age at time of PMSC testing, intervention group and mothers’ group attended. ^1^ Ref. (lowest tertile); ^2^ Tertiles were capped as follows: time spent with other children/older children and time spent free to move about capped at 14 h per day (mean waking time for child of this age); time outdoors and time spent being physically active with mum capped at 7 h/day (half of waking day).

**Table 4:** Correlates of 5-year-old children’s perceived motor skill competence (PMSC).

Variable	Univariate Models Sample Size (*n*)	Univariate Models β (95% CI)	
**Child behaviours ^2^**			
Time spent being physically active with mum	280	Medium High	0.35 (−1.81, 2.50) ^1^ 1.18 (−1.00, 3.37)
Time spent ‘free to move around’	266	Medium High	−1.02 (−3.23, 1.22) ^1^ −0.74 (−2.95, 1.47)
Time spent with other children of a similar age	269	Medium High	0.11 (−2.13, 2.35) ^1^ −0.07 (−2.31, 2.17)
Time spent with older children	264	Medium High	−1.08 (−3.31, 1.14) ^1^ −1.56 (−3.70, 0.58)
Time spent outside	293	Medium High	−0.24 (−2.35, 1.87) ^1^ 45 (−0.65, 3.56)
**Maternal beliefs**			
Maternal physical activity knowledge	284		−0.65 (−3.26, 1.96)
Maternal physical activity views	284		−0.41 (−2.34,1.53)
Maternal physical activity optimism	284		1.09 (−0.62, 2.79)
Maternal physical activity self-efficacy	284		−0.53 (−2.11,1.05)
**Parental behaviours**			
Parental facilitation of physical activity	273		0.01 (−0.05, 0.08)
**Home environment**			
Ball skills equipment in home	273		−0.06 (−0.43, 0.32)
Play equipment in home	273		0.06 (−0.07, 0.20)
**Community environment**			
Sport participation (Y/N)	300		Ref. (No) −0.01 (−2.44, 2.41)
Dance/Gymnastics (Y/N)	300		Ref. (No) −0.22 (−2.34, 1.91)
Swimming (Y/N)	300		Ref. (No) 0.23 (−1.62, 2.08)

^1^ Ref. (lowest tertile); ^2^ Tertiles were capped as follows: time spent with other children/older children and time spent free to move about capped at 14 h per day (mean waking time for child of this age); time outdoors and time spent being physically active with mum capped at 7 h/day (half of waking day).

## Data Availability

The dataset analysed for the current study is not publicly available due to ethical restrictions related to the consent given by participants at the time of study commencement. An ethically compliant de-identified dataset may be made available by the corresponding author on reasonable request and upon approval by the Deakin University Human Research Ethics Committee.
